# Defining Brain Fog - A Transdiagnostic Narrative Review

**DOI:** 10.1192/j.eurpsy.2025.1226

**Published:** 2025-08-26

**Authors:** P. Denno, A. Hampshire

**Affiliations:** 1 Imperial College London; 2 Kings College London, London, United Kingdom

## Abstract

**Introduction:**

The term brain fog is increasingly used by patients and researchers, but is poorly-defined. Understanding the symptom is important, as it is highly-prevalent, and linked to poor quality of life, and social and occupational disability. Furthermore, it is reported in a broad and seemingly unrelated set of conditions including COVID, menopause, hypothyroidism, traumatic brain injury (TBI), chronic fatigue syndrome (CFS/ME), fibromyalgia, and systemic lupus erythematosus (SLE). Transdiagnostic similarities may indicate common mechanisms or therapeutic targets, and opportunities for translation of research findings.

**Objectives:**

We aimed to review research characterising brain fog across diagnoses and summarise findings on phenomenology, definitions, objective cognitive measures, and neurobiological correlates. We aimed to highlight transdiagnostic commonalities and differences, and make recommendations on terminology and future research.

**Methods:**

We conducted a narrative review of biomedical research into brain fog. We chose a non-systematic approach due to the fragmentary nature of the literature and our exploratory aims.

**Results:**

Brain fog is associated with cognitive symptoms - predominately difficulties with attention, memory, and language, contributing to a subjective “fog” or lack of “mental clarity”. It overlaps with fatigue, and psychiatric symptoms, including anxiety, depression, and dissociation. It is chronic but often transient or variable. There are many transdiagnostic similarities, but research conducting direct comparisons is lacking. We argue that transdiagnostic commonalities in brain fog must arise at one of 3 levels - ambiguous language, common cognitive mechanisms, or common neurobiology. Neurobiological correlates appear heterogenous. Objective cognitive findings are mixed, between mild deficits in attention and memory and no deficits. Evidence for correlation between subjective and objective symtpoms is mixed. Many studies find objective deficits are mediated by associated symptoms such as fatigue, depression, or anxiety.

**Conclusions:**

We suggest researchers avoid the term brain fog in favour of clearly-defined terms where possible. While brain fog appears to refer to a broad range of phenomena, it captures a characteristic assocation of fatigue, cognitive and affective symptoms, and mild objective deficits across diagnoses. Brain fog appears to overlap substantially with mental fatigue. Further research is needed, including direct transdiagnostic comparisons. Measures should include high-precision cognitive batteries, as well as measures of affect (e.g., GAD / PHQ9), fatigue (e.g., FAS), and metacognition, to enable the role of non-cognitive factors to be assessed and compared across conditions.

**Disclosure of Interest:**

None Declared

